# Perceived social support functions as a resilience in buffering the impact of trauma exposure on PTSD symptoms via intrusive rumination and entrapment in firefighters

**DOI:** 10.1371/journal.pone.0220454

**Published:** 2019-08-02

**Authors:** Jong-Sun Lee

**Affiliations:** Department of Psychology, Kangwon National University, Chuncheon, Republic of Korea; Yale University, UNITED STATES

## Abstract

Resilience has been highlighted as a pivotal factor in overcoming the detrimental impact of trauma. The present study tests a resilience model of trauma using risk (rumination, emotion regulation, and entrapment) and protective (perceived social support) factors in a sample of firefighters who are at heightened risk for post-traumatic stress disorder, using a cross-sectional design. Specifically, the present study focuses on perceived social support as a resilience factor against PTSD symptoms, in response to trauma exposure. The sample included 545 firefighters from six large cities in Korea, who completed the following self-report questionnaires: Life Event Checklist, Event-related Rumination Inventory, Emotion Regulation Questionnaire, Entrapment scale, Impact of Event Scale-Revised, and Duke-UNC Functional Social Support Questionnaire. Exposure to traumatic events indirectly affected PTSD symptoms via intrusive rumination, emotional regulation, and perceptions of entrapment. Additionally, the mediating effects of intrusive rumination and perceptions of entrapment were moderated by perceived social support. That is, firefighters with high levels of perceived social support reported lower severity of rumination and PTSD symptoms. These findings suggest that perceptions of social support may operate as a resilience factor in buffering the effects of trauma on PTSD symptoms. Perceived social support interacts with intrusive rumination and perceptions of entrapment, thereby resisting the development of PTSD symptoms.

## Introduction

Firefighters are at risk for post-traumatic stress disorder [1] due to repeated exposure to traumatic events. The estimated prevalence rate of PTSD among firefighters ranges from 18% to 22% [2, 3], which is three times higher than the general population[4]. However, the empirical research on PTSD in firefighters is limited, despite a growing interest in PTSD in firefighters as well as other vulnerable populations, such as combat-veterans or sexual abuse victims. As firefighters work in the front line of disasters, they are a particularly vulnerable group for PTSD, which suggests that research and intervention related to PTSD in firefighters should be a national priority.

Recently, there has been a growing body of resilience research in the context of PTSD. Resilience theory suggests that resilience requires the exposure to risk factors, such as adversities or traumatic events [5]. Firefighters are frequently exposed to traumatic events. Further, the protective resilience model stresses that resilience includes not only risk but also protective factors simultaneously. This is because resilience refers not only to a resilient outcome itself, but to the process of overcoming the risk [6]. As such, a protective model of resilience explores the interactions between risk and protective factors, thereby reducing negative consequences and promoting positive ones. This approach enables the researcher to identify how protective factors interact with risk factors to generate specific outcomes, which has been considered as a key component for future studies in this area. Another point that should be considered in the resilience model is that there could be multiple risk factors. Individuals who have experienced traumatic events have actually been exposed to multiple risk factors. In addition, resilience is, by definition, not limited to individual traits, but includes social and environmental factors. The present study aims to test the protective model of resilience proposed by Fergus and Zimmerman (see [6] for a review) in the context of PTSD in firefighters, using a moderated mediation model that tests the interaction between risk and protective factors in the same model.

All individuals who are exposed to traumatizing incidents do not develop PTSD. Following trauma exposure, some individuals develop PTSD that considerably undermines their ability to function in daily life, whereas others recover from the impact of trauma within a short period, thereby being protected from the development of PTSD. What factors confer psychological variability for adapting to trauma? Resilience factors may be a pivotal predictor in determining individual difference in posttraumatic adjustment. For example, using a moderated mediation analysis, a previous study found that under the same level of trauma experience, firefighters with high levels of resilience were protected from both the direct and indirect impacts of trauma exposure on PTSD via perceived stress, relative to those with low levels of resilience [7]. As Fergus and Zimmerman indicated [6], there is increasing evidence that social support is also an important resilient factor in PTSD [8]. For example, social support appeared to buffer the impact of trauma exposure in sexual abuse victims [9], war veterans [10], and earthquake survivors [11]. Previous research identified several types of social support and, in particular, perceived (functional) and received social support have been considered in terms of the context of PTSD. Perceived social support is theoretically and empirically distinct from received social support. Perceived social support is based on the perception of how beneficial the received social support is in meeting emotional or instrumental needs, and received social support is an objective measurement of the frequency of social interactions, along with instrumental and material support [12]. That is, received social support that is actually offered to participants is different from one’s perceptions that support is available or will be available in the future. Given that the relationship between stress and stress-reaction processing, a stressor triggers a stress reaction or symptoms if the coping resources are perceived as insufficient for handling the given situation, an emphasis can be placed on the importance of “cognitive appraisal” on stress reaction and prediction of stress related symptoms. [13]. Interestingly, a meta-analysis found that perceived social support reported by first responders (e.g., firefighters, police officers) showed higher effect sizes than received social support in moderating the impact of traumatic events [14]. It might be that the effectiveness of social support varies by one’s subjective perception of whether the support satisfies one’s needs. Indeed, a recent study found that perceived social support from supervisors, coworkers, and family/friends appeared to be a protective factor in mitigating PTSD symptoms in female firefighters [15]. It would be interesting to investigate whether such results would be replicated in male firefighters, especially when using a resilience model.

On the other hand, the relationship between trauma exposure and the severity of PTSD symptoms is not direct. Furthermore, previous studies have explored several psychological factors that underpin this relationship. Maladaptive coping strategies such as intrusive rumination are one plausible risk factor in the response to traumatic events. For example, although intrusive thoughts are known to become a normative thought process in response to trauma, persistent intrusive thoughts are a significant risk factor for PTSD symptoms in combat veterans and university students [16–18]. Therefore, it might be logical to hypothesize that intrusive rumination would be a significant mediator of PTSD symptoms after trauma exposure in firefighters. Other maladaptive coping strategies, such as suppression of negative emotion, would also affect the development and maintenance of PTSD symptoms [19]. Given that firefighters share “macho culture”, it is not easy for them to express negative feelings after the exposure of trauma, thereby facilitating suppression rather than expression in regulating their emotion. Indeed, emotional suppression appeared to have a positive relationship with high levels of physical and psychological stress symptoms in ambulance workers and firefighters [20]. However, as both studies did not directly assess PTSD symptoms, the hypothesis regarding whether emotional suppression would affect PTSD symptoms remains to be answered in future research. A sense of entrapment might be a potential risk factor for PTSD symptoms after trauma for firefighters [21]. Entrapment relates to a sense that comes from external circumstances or internal thoughts and feelings. A sense of entrapment involves psychological processes related to one’s subjective perception of his or her circumstances, feelings, and thoughts as being uncontrollable and inescapable [21]. Such perceptions emerge in various situations such as difficult jobs or relationships, health problems, distressing emotions, and a lack of resources. A sense of entrapment is known to be related to stressful events that are incessant, ongoing and chronic. Stressful events for firefighters are ongoing and chronic, which may engender feelings of inescapable and uncontrollable stress, thereby bringing about a sense of ongoing threat, which is central to the persistence of PTSD symptoms [19]. Previous studies have suggested that the perception of entrapment in response to a traumatic stress situation is an important mediator underlying the association between trauma and PTSD symptoms. A sense of entrapment often occurs in high-stress situations in which escape is motivated, but blocked or prevented, which would subsequently engender high levels of stress symptoms [22]. Indeed, prior studies have found that the perception of entrapment mediated the relationship between nightmares and suicidal behaviors [23], and between negative self-appraisal and suicidal behavior [24] in those with PTSD symptoms. A recent meta-analysis showed that there is a large effect size of entrapment and PTSD [25]. However, the mediating effect of the perception of entrapment in the relationship between trauma exposure and PTSD symptoms has not yet been directly tested in firefighters. Exposure to trauma in firefighter is not avoidable event as it is an important part of their job duty. It might be plausible to predict that the perception of entrapment would be a significant risk factor in the development of PTSD symptoms in firefighters.

Understanding the role of perceived social support as a resilience factor for overcoming the detrimental impact of trauma on PTSD symptoms via intrusive rumination, emotion regulation, and entrapment is of great importance. Perceived social support, that is, the perceptions that support from others would be available, useful, and satisfying if needed, is known to be a protective factor for one’s cognitive, emotional, and behavioral adjustment during times of stress[26]. Main effects of such perceived social support on mental health might occur when individuals regulate their negative thoughts and emotions. Indeed, there is evidence that the perception of social support is significantly associated with ruminative thoughts [27, 28] and appears to mitigate the detrimental effect of state rumination on negative affects [29]. Furthermore, the social cognitive theory posits that social expectations are crucial in one’s emotion regulation. Positive expectations regarding others’ availability in case of need are itself a main self-regulating mechanism by making emotional distress more tolerable and manageable [30]. Perceived social support also promotes a sense of personal control [31], which may presumably interact with a sense of entrapment that is related to a concept of subjective controllability to one’s inner feelings and environments. However, empirical evidence is yet to be explored. Using a moderated mediation analysis, the present study aims to test a potential hypothesis that social support might contribute to attenuate PTSD symptoms by decreasing ruminative thoughts, emotional suppression, and a sense of entrapment. Answering this hypothesis offers an indirect theoretical account of how social support functions resilience in overcoming risk factors after traumatic adversities.

Taken together, the present study aims to investigate a resilience model for firefighters, which includes both a protective factor (perceived social support) as well as risk factors (ruminative thoughts, emotional suppression, and sense of entrapment). Using moderated mediation statistics, the present study aims to identify the specific range within which the moderator (perceived social support) would have a statistically significant conditional indirect effect. This allows us to detect who may be more resistant or susceptible to PTSD symptoms with the same levels of trauma exposure, which would in turn help in developing an individually tailored intervention program. Thus, the hypotheses of the present study are the following: 1) trauma exposure would be positively associated with the risk of PTSD symptoms via the mediating effects of intrusive rumination, emotional suppression, and sense of entrapment; and 2) perceived social support moderates the indirect effect of trauma exposure on PTSD symptoms via intrusive rumination, emotional suppression, and sense of entrapment.

## Materials and methods

### Participants and procedure

A total of 672 firefighters, including first-responders and rescuers, from six large cities in 00 initially responded to the online survey used in the present study; however, 127 firefighters were excluded from the final analyses due to missing data, low reliability (e.g., the same response on all items in a questionnaire, completion of less than half of the questionnaires), and refusal to participate. Thus, the final sample included a total of 545 firefighters whose informed written consent was obtained (see Table 1 for demographic characteristics). Firefighters that were included and excluded in the final analysis did not differ in age, t(601) = 1.19, ns, total duration of employment, t(603) = 0.53, ns, gender, χ ^2^ (1) = 1.05, ns, marital status, χ ^2^ (3) = 242, ns, education, χ ^2^ (7) = 2.53, ns, or type of duty, χ ^2^ (4) = 9.72, *p* = .98. Information about the study was first delivered via the fire stations’ internal email system. If willing to participate, participants were asked to open the online survey link provided in the email and complete a series of questionnaires, including the Life Event Checklist, the Event Related Rumination Inventory-intrusive rumination subscale, the Emotion Regulation Questionnaire- suppression subscale, the Entrapment Scale, the Functional Social Support Questionnaire, and the Impact of Event Scale-Revised. All data were assigned numeric identifiers to ensure the confidentiality of participants and stored accordingly. Kangwon National University Institutional Review Board approved this study (KNUIRB-2015-06-001-003).

**Table 1:** Number of different traumatic events experienced (N = 545).

Traumatic Events	Happened to me N(%)	Witnessed it N(%)	Learned it N(%)	Part of my job N(%)
· Natural disaster	75(13.8)	180(33.0)	75(13.8)	185(33.9)
· Fire or explosion	52(9.5)	199(36.5)	32(5.9)	291(53.4)
· Transportation	137(25.1)	196(36.0)	45(8.3)	278(51.0)
· Serious accident at work, home, or during recreational activity	61(11.2)	92(16.9)	82(15.0)	133(24.4)
· Exposure to toxic substance	13(2.4)	35(6.4)	96(17.6)	117(21.5)
· Physical assault	132(24.2)	77(14.1)	92(16.9)	104(19.1)
· Assault with a weapon	27(5.0)	40(7.3)	100(18.3)	70(12.8)
· Sexual assault	2(.4)	12(2.2)	79(14.5)	20(3.7)
· Other unwanted or uncomfortable sexual experience	16(2.9)	7(1.3)	50(9.2)	16(2.9)
· Combat or exposure to a war-zone	7(1.3)	4(.7)	24(4.4)	8(1.5)
· Captivity		3(.6)	21(3.9)	8(1.5)
· Life-threatening illness or injury	25(4.6)	50(9.2)	64(11.7)	77(14.1)
· Severe human suffering	41(7.5)	35(6.4)	51(9.4)	73(13.4)
· Sudden violent death	5(.9)	123(22.6)	60(11.0)	215(39.4)
· Sudden accidental death	132(24.2)	86(15.8)	84(15.4)	95(17.4)
· Serious injury, harm, or death you caused to someone else	11(2.0)	11(2.0)	19(3.5)	23(4.2)
· Any other very stressful event or experience	88(16.1)	55(10.1)	44(8.1)	166(30.5)
Number of endorsed traumatic event				
1	111(20.4)	87(16.0)	105(19.3)	53(9.7)
2	66(12.1)	69(12.7)	56(10.3)	60(11.0)
3	70(12.8)	62(11.4)	45(8.3)	62(11.4)
4	31(5.7)	35(6.4)	29(5.3)	54(9.9)
Above 5	39(7.2)	99(18.3)	77(14.1)	176(32.3)

### Assessments

Demographic information, such as gender, age, level of education, marital status, and type of duty, was obtained using a self-report questionnaire.

#### The Life Event Checklist (LEC) - 5

The number of traumatic events experienced by a participant was assessed using the LEC-5-standard version [32]. The present study used the validated Korean version of the LEC, which added minimal changes to the LEC-5 (item 5, “Sudden, unexpected death of someone close to you” was changed to “Sudden accidental death”, and response category of “Part of my job: was added) [33]. LEC-5 consists of 17 items describing traumatic events such as natural disasters, exposure to toxic substances, and assault with a weapon. For each of the traumatic events, participants were asked to select one of the following options: “happened to me,” “witnessed it,” “learned about it,” “part of my job”, “not sure,” and “doesn’t apply.” The frequency of the types of traumatic events for which the response “happened to me,” “witnessed it,” “learned it,” and “part of my job” was selected and summed in the final analyses. Table 1 shows the average number of different traumatic events experienced.

#### Event-Related Rumination Inventory (ERRI)

Intrusive rumination was measured using the K version of the ERRI, validated by Ahn, Joo [34], and originally developed by Cann, Calhoun [18]. The ERRI assesses the frequency of both intrusive and deliberate rumination and consists of 10 items each for both kinds of rumination. The present study included the intrusive rumination scale. Participants rated each item on a 4-point Likert scale ranging from 0 (“Not at all”) to 3 (“Often”). Total scores range from 0 to 30, with higher scores reflecting greater intrusive rumination.

#### Emotion Regulation Questionnaire (ERQ)

Emotional suppression was measured using the K version of the Emotion Regulation Questionnaire (ERQ) translated by Juna Byun and her team (downloaded from http://spl.stanford.edu/pdfs/ERQ/Korean.pdf). The ERQ was originally developed by Gross and John (2003), and includes two subscales, reappraisal and suppression. The present study included the suppression subscale in the final analysis, which included 5 items rated on a 7-point Likert scale ranging from 1 (“Strongly disagree”) to 7 (“Strongly agree”). The total scores range from 0 to 35, with higher scores indicating greater suppression.

#### Entrapment Scale (ES)

Sense of entrapment was assessed using the Entrapment Scale (ES) developed by Gilbert & Allan (1998). The present study included the validated Korean version of the ES [35]. This scale considers the perception of circumstances (external entrapment) and internal thoughts or feelings (internal entrapment) to be uncontrollable and inescapable. The 16-item questionnaire uses a five-point Likert scale ranging from 1 (“Not at all like me”) to 5 (“Extremely like me”). Total scores range from 0 to 80, with higher scores indicating greater entrapment.

#### Impact of Event Scale-Revised (IES-R)

PTSD symptoms were assessed using the Impact of Event Scale-Revised (IES-R; Wiess, 2007). Horowitz, Wilner [36] originally developed the scale to evaluate posttraumatic stress symptoms, and Weiss [37] revised it by adding hyperarousal symptoms along with intrusion, avoidance, and numbness. The IES-R consists of 22 items rated on a five-point Likert scale ranging from 0 (“Not at all”) to 4 (“Extremely”). Total scores ranged from 0 to 88, with higher scores reflecting more posttraumatic symptoms.

#### Duke_UNC Functional Social Support Questionnaire (FSSQ)

Perceived social support was measured using the Duke_UNC Functional Social Support Questionnaire (FSSQ), which was developed by Broadhead, Gehlbach [38]. This measure assesses one’s subjective perception of confidant and emotional support and consists of eight items (confidant support: five items, emotional support: three items) rated on a 5-point Likert scale ranging from 1 (“Much less than I would like”) to 5 (“As much as I would like”). Total scores range from 1 to 40, with higher scores indicating higher perceived social support.

## Statistical analyses

**Fig 1:**
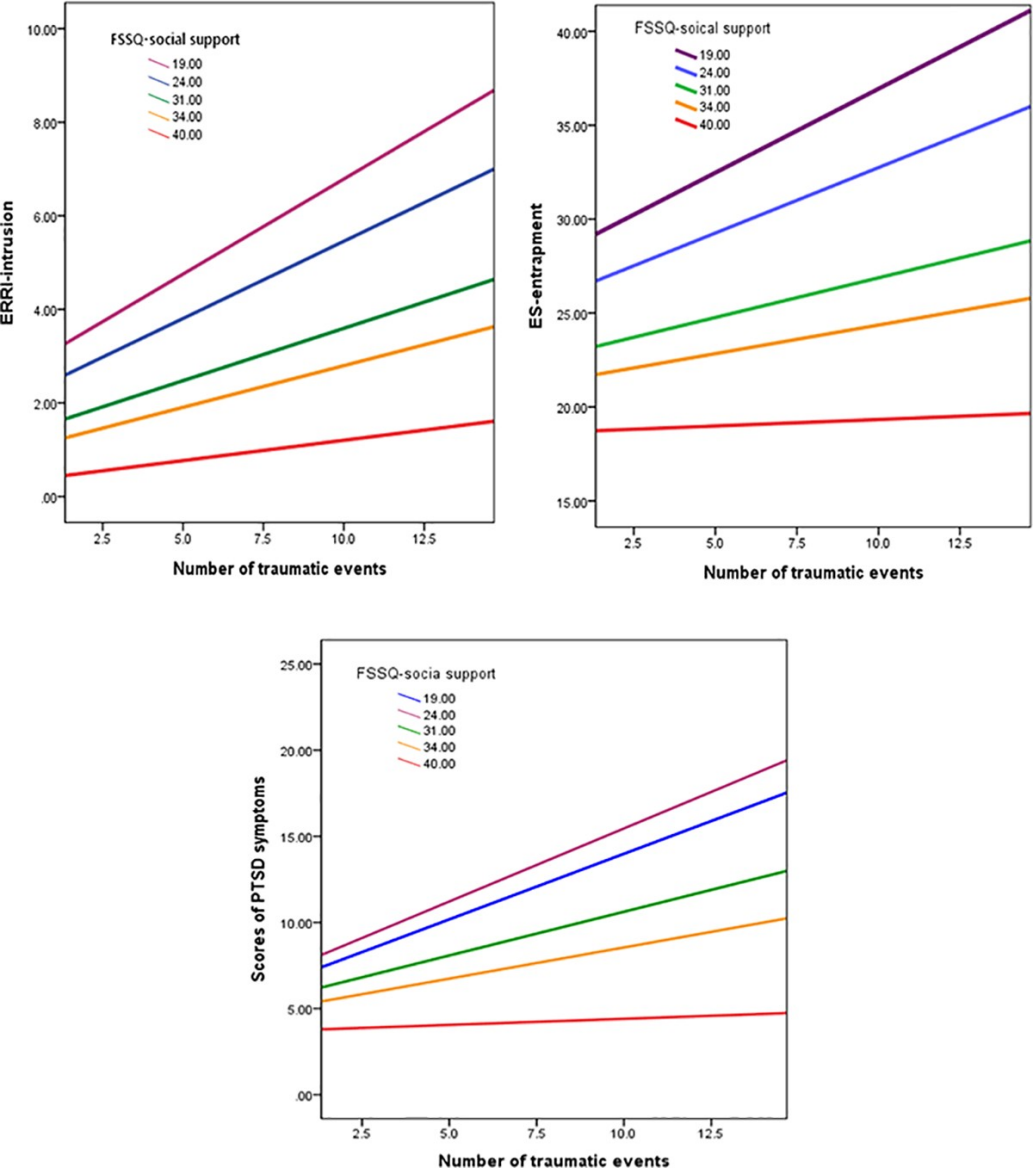
The number of traumatic stress events as a function of perceived social support. This interaction predicts event-related intrusive rumination, entrapment, and PTSD symptoms. The slope indicates that as the number of traumatic events increases, event-related intrusive rumination, entrapment, and PTSD symptoms also increase. However, the slope is increasingly reduced as the level of perceived social support increases. Fig 1 caption followed the descriptions of the previous work [7].

Descriptive and correlational analyses of the six variables (traumatic events, event related intrusive rumination, suppressive emotion regulation, entrapment, posttraumatic symptoms, and social support) were performed using SPSS 21.0 Software. Moderated mediation analyses were performed using SPSS PROCESS macro [39]. The PROCESS analysis included one independent variable (i.e., traumatic events), three mediators (i.e., event-related intrusive rumination, suppressive emotion regulation, entrapment), one moderator (social support), and one dependent variable (i.e., PTSD symptoms (Fig 1). The variables were mean-centered prior to the analysis. The number of bootstrap samples for the bias-corrected bootstrap confidence intervals (CIs) was 10000. The normality of the data for each variable was checked using skewness and kurtosis values, along with a visual inspection of histograms and box plots (Table 2). The present study used Model 59 in PROCESS to test the moderated mediation analysis. The conditions to confirm the moderated mediation followed the guideline provided by Preacher, Rucker [40]; the specific conditions are the same as in the previous study [7].

**Table 2:** Characteristics of the sample (N = 545).

Characteristics of the sample	%	N
Age (N = 537, missing = 8)		
20–29 years	12.5%	67
30–39 years	42.0%	229
40–49 years	31.9%	174
50–59 years	12.1%	66
60–69 years	0.2%	1
Gender (N = 545)		
Male	94.1%	513
Female	5.9%	32
Marital status (N = 544, missing = 1)		
Married	71.7%	391
Single	26.6%	145
Divorced	1.1%	6
Separated	0.4%	2
Education (N = 531, missing = 14)		
Elementary school	0.2%	1
Middle school	0.9%	5
High school	23.1%	126
College/university	71.2%	388
Graduate school	2.0%	11
Type of duty (N = 536, missing = 9)		
Suppression of fire	25.9%	141
Driving	21.8%	119
First respond	29.7	162
Rescue	14.7%	80
Not specified	6.2%	34
Average duration of employment	129.36 months	539

## Results

### Demographic characteristics of the sample

The demographic characteristics of participants are presented in Table 2.

### Preliminary correlation analyses

Correlations of the six variables are presented in Table 3. The number of traumatic events was positively associated with PTSD symptoms, intrusive rumination, emotional suppression, and entrapment, and inversely correlated with perceived social support. PTSD symptoms were positively associated with intrusive rumination, emotional suppression, and entrapment, and inversely correlated with perceived social support. Notably, the correlation coefficient between intrusive rumination (mediator) and PTSD symptoms (dependent variable) appeared to be .79. However, the event-related intrusive rumination that was assessed in the present study is composed of different constructs from PTSD symptoms (e.g. “re-experiencing”) in that it emerges in response to positive as well as negative life events, and is even considered to be a normal psychological process in the face of significant experiences [18]. Indeed, the intrusive factor explained the variance of deliberate rumination that cannot be accounted for PTSD symptoms assessed with the IES-R [18]. Finally, prior to moderated mediation, we checked multicollinearity between predictor variables using Tolerance and VIF. All values of Tolerance were above 0.1, and those of VIF appeared to be under 10, therefore, we further proceeded to analyze moderated mediation.

**Table 3:** Mean, standard deviation, and correlation between the main variables (N = 545). PTSD = post-traumatic stress disorder.

Variables	1	2	3	4	5	6
1. Trauma	1					
2. PTSD symptoms	.30***	1				
3. Intrusive rumination	.30***	.79***	1			
4. Suppression	.10*	.26***	.25***	1		
5. Entrapment	.30***	.58***	.61***	.29***	1	
6. Social Support	-.15***	- .35***	- .34***	- .16***	- .45***	1
Mean	9.04	10.55	3.50	13.53	27.26	29.35
Standard deviation	5.69	12.74	5.21	4.95	12.41	7.10
Skewness	1.26	1.88	1.71	- .02	.93	- .49
Kurtosis	2.75	4.27	2.61	.09	.06	-.17

Note.

*** *p* < .001

* *p* < .05

### Moderated mediation

First, the mediator variable model (Table 4) indicated that the number of traumatic events experienced by the firefighters was positively correlated with their levels of intrusive rumination, emotional suppression, and entrapment. The level of perceived social support reported by the firefighters was inversely correlated with the levels of intrusive rumination, emotional suppression, and entrapment. The interactions between the number of traumatic events and perceived social support in the relationship between the number of traumatic events and intrusive rumination, as well as those between the number of traumatic events and entrapment, respectively, were significant. However, the interaction effect between the number of traumatic events and perceived social support in the relationship between the number of traumatic events and emotional suppression was not significant.

**Table 4:** Moderated mediation analyses (N = 545).

*Mediator variable model*						
Outcome variable	**Intrusive rumination**		Suppression		**Entrapment**	
	B (*SE*)	*t*	B (*SE*)	*t*	B (*SE*)	*t*
Trauma	.24(.04)	6.73***	.07(.04)	2.00*	.53(.08)	6.55***
Social Support	- .22(03)	-7.74***	-.11(.03)	-3.71**	-.72(.07)	-11.01***
Trauma x Social support	-.02(.01)	-3.23**	-.01(.01)	-1.01	-.04(.01)	-3.09*
*Dependent variable model*						
	Outcome variable: PTSD symptoms					
	B (*SE*)			*t*		
Intrusive rumination	1.61(.09)			18.88***		
Suppression	.15(.07)			2.08*		
Entrapment	.10(.04)			2.56**		
Trauma	.12(.06)			1.96		
Social support	-.10(.05)			-1.95		
A	-.01(.01)			-.76		
B	-.01(.01)			-.90		
C	-.00(.01)			- .52		
D	-.01(.01)			-1.09		
Conditional indirect effect						
Mediator	Moderator: social support	Indirect effect (SE)		LL 95%CI	UL 95%CI	
Intrusive rumination	-10.3541(10th percentile)	.6934(.1502)		.4245	1.0189	
	-5.3541(25th percentile)	.5411(.0990)		.3584	.7530	
	1.6459(50th percentile)	.3401(.0652)		.2201	.4786	
	4.6459(75th percentile)	.2584(.0676)		.1411	.4072	
	**10.6459(90th percentile)**	**.1029(.0910)**		**-.0510**	**.3051**	
Entrapment	-10.3541(10th percentile)	.1133(.0627)		.0117	.2503	
	-5.3541(25th percentile)	.0795(.0344)		.0237	.1550	
	1.6459(50th percentile)	.0178(.0111)		.0106	.0851	
	4.6459(75th percentile)	.0165(.0021)		.0015	.0734	
	**10.6459(90th percentile)**	**.0157(0.0119)**		**-.0068**	**.0659**	

*Note*.

*** *p* < .001

** *p* < .01

* *p* < .05

A: Intrusive rumination x Social support

B: Suppression x Social support

C: Entrapment x Social support

D: PTSD symptoms

x Social support

**Fig 2:**
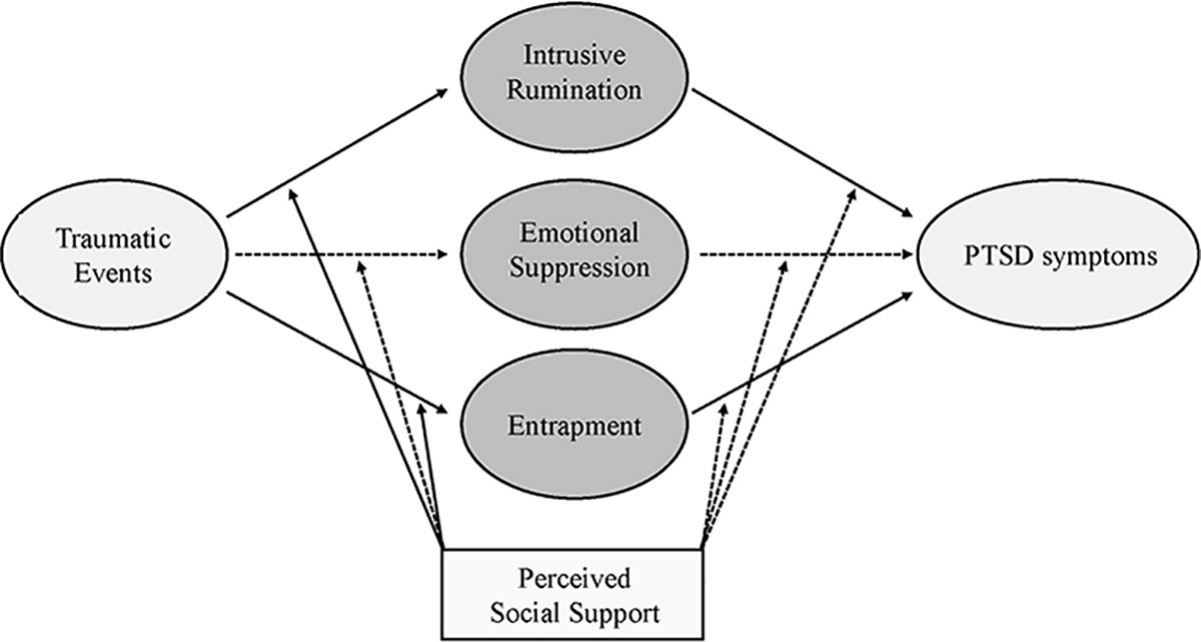
The final moderated mediation model. The indirect link associating traumatic events and PTSD symptoms via event-related intrusive rumination and entrapment is moderated by perceived social support. The dotted line indicates a non-significant pathway in the association between traumatic events and PTSD symptoms.

Similar to the results of the previous work [7], the conditional indirect effect of traumatic events on PTSD symptoms through intrusive rumination and entrapment differed according to the degree to which a subject perceived social support (Table 3). More specifically, the conditional indirect effect was weaker in the upper 10^th^ percentile of firefighters, whereas it was significant in up to the 75^th^ percentile of firefighters (a bootstrapped 95% CI did not include zero). These results imply that the greater the number of traumatic events experienced by the firefighters, the greater the level of their intrusive rumination and entrapment, thereby making them vulnerable to the development of PTSD symptoms (up to the 75^th^ percentile). However, firefighters with a high level of perceived social support (upper 10^th^ percentile) may be protected from the risk of developing PTSD symptoms via resistance to intrusive rumination and entrapment (Fig 1). Fig 2 shows the final moderated mediation model.

## Discussion and conclusion

The present study aimed to explore possible pathways of risk for the development of PTSD in firefighters who are regularly exposed to traumatic events. Based on a resilience model and using a moderated mediation analysis, the current study sought to investigate the interaction effects of risk and protective factors underlying trauma exposure and PTSD symptoms. The key findings are as follows: firstly, trauma exposure indirectly affected the severity of PTSD symptoms via intrusive rumination, emotional suppression, and entrapment. Secondly, the mediating effects of intrusive rumination and entrapment, but not emotional suppression, were moderated by perceived social support.

Full mediating effects of intrusive rumination, emotional suppression, and entrapment were found in the relationship between trauma exposure and PTSD symptoms; thus, hypothesis 1 was fully supported. These results imply that the effects of trauma through the pathways of the increased levels of intrusive rumination, emotional suppression, and entrapment influence PTSD symptoms. That being said, the more the firefighters are exposed to traumatic events, the more they report experiencing intrusive rumination, entrapment, and emotional suppression, which in turn confers vulnerability for the development of PTSD symptoms. These results are in line with prior findings that indicate intrusive rumination as a pivotal risk and mediating factor for PTSD symptoms in combat veterans and university students [17, 41]. Our results expand the field of existing research by demonstrating that intrusive rumination mediates the relationship between trauma exposure and PTSD symptoms in firefighters. Previous studies have also noted that suppression of negative emotion is positively associated with physical and psychological stress reactions in ambulance workers and firefighters [20]. However, none of these studies explored the relationship between emotional suppression and PTSD symptoms. Our study is the first to demonstrate that trauma exposure in firefighters indirectly affects PTSD symptoms via emotional suppression. Given the prevalence of “macho culture” among firefighters, they may be more likely to use suppression rather than expression as a strategy for regulating their negative emotions, which consequently increases their vulnerability to PTSD symptoms. On the other hand, our findings demonstrate the full mediating effect of entrapment in the pathway between traumatic events and PTSD symptoms in firefighters. For firefighters, trauma is not an isolated and time-limited event, but rather a series of ongoing events. Although firefighters might wish to escape from exposure to traumatic events, it is challenging to do so when rushing to the scene of traumatic accidents is a requirement of their job. In this sense, the perception of entrapment might be a pivotal risk factor related to the development and maintenance of PTSD symptoms in firefighters. Thus far, entrapment has been considered as an important risk factor for depression and anxiety (see [42] for a review). The results of the current study expand on these findings and suggest that the perception of entrapment might be an important psychological factor underlying the relationship between trauma exposure and PTSD symptoms.

Notably, not all firefighters develop PTSD symptoms through an increase of intrusive rumination and perception of entrapment; As shown in Fig 1, with the same number of traumatic events, perceived social support appeared to be negatively associated with intrusive rumination, perception of entrapment, and PTSD symptoms. That is, firefighters with high levels of perceived social support (upper 90^th^ percentile) reported lower scores of PTSD symptoms, intrusive rumination and perception of entrapment, whereas firefighters in the lower 75^th^ percentile of perceived social support were more likely to report higher scores of PTSD symptoms. Given that intrusive rumination and entrapment in the present study were included as mediators, these results offer indirect evidence that perceived social support might contribute to attenuate PTSD symptoms by intervening intrusive rumination and sense of entrapment. However, given that the present research design is cross-sectional, the results should be interpreted cautiously. To confirm the resilient function of perceived social support on intrusive rumination and sense of entrapment in a causational manner, a longitudinal or experimental design should be included in a future study. These results are in line with a resilience model of social support, which shows that social support is an important resilience factor against PTSD symptoms and heightened suicidal behaviors in individuals with PTSD symptoms [15, 43]. Our findings highlight the role of perceived social support as a key resilience factor in buffering the effects of intrusive rumination and perception of entrapment that lead to PTSD symptoms after exposure to repeated traumatic events.

### Limitations

Several limitations should be noted. First, a cross-sectional design was used in the current study, which limits the interpretation of causal relationships between the observed variables. For example, we are not able to clarify the casual relationships between emotional suppression and intrusive rumination and the exposure to a traumatic event. A longitudinal design starting at the stage of recruitment for firefighters or experimental designs could clarify the causal relationships between the variables. Second, firefighters in the present study completed online self-reported questionnaires and may have over- or under- reported their PTSD symptoms. Clinician-administered interviews regarding PTSD symptoms might reflect more accurate information; thus, a future study is warranted to include more objective measurements. Third, the model proposed in the current study is based on Korean firefighters. It is unknown if the resilience model in the present study would be applicable to those in other countries, suggesting that this study should be replicated in different populations. Fourth, the ERQ measurement used in the present study has two subscales, namely, reappraisal and suppression, and we only included suppression. However, suppression did not interact with perceived social support. In a future study, it might be interesting to examine whether reappraisal factors would mediate the relationship between trauma exposure and PTSD symptoms, and finally interact with perceived social support. Reappraisal might also be a moderator as reappraisal is considered to be one of the main intervention targets in Cognitive Behavioral Therapy. Finally, the majority of the sample comprised male firefighters, which precludes the generalization of the results of the current study. A future study with a large sample of female participants is warranted in order to test whether the results of the present study can be generalized to women.

Despite these limitations, our study has several strengths both empirically and clinically. We identified three key mediating factors including intrusive rumination, emotional suppression, and perceptions of entrapment. Among these, intrusive rumination and perceptions of entrapment appeared to interact with perceived social support. That is, one’s perceptions about social support may be a key resilience factor for acting as a buffer for firefighters against increased intrusive rumination and perceptions of entrapment, thereby reducing the high risk of developing PTSD symptoms. Future studies should also address how other factors function to promote resilience in the link between trauma exposure and PTSD symptoms through emotional suppression.

## Supporting information

S1 Data Dataset of study. (SAV)
